# Regional anaesthesia for district hospitals and clinics

**DOI:** 10.4102/safp.v66i1.5872

**Published:** 2024-06-28

**Authors:** Michele Torlutter

**Affiliations:** 1 Department of Family Medicine, Faculty of Health Sciences, University of the Witwatersrand, Johannesburg, South Africa

**Keywords:** low-resource countries, regional anaesthesia, ultrasound-guided nerve blocks, anaesthesia safety

## Abstract

Pain is a common reason that patients seek care in the emergency department (ED). Regional anaesthesia in the form of nerve blocks provides an excellent alternative to traditional forms of analgesia, and may be superior in managing musculoskeletal pain compared to opioids. Adequate pain management improves patient satisfaction, facilitates examination and minor procedures, and allows for earlier and safe discharge. In low resource settings this modality is underutilised due to lack of trained providers and/or support from specialised services, shortages of equipment, and lack of context-sensitive guidelines. Advances in ultrasound guided regional anaesthesia has the potential to improve access to safe and reliable anaesthesia. It is often not accessible or an active part of training even for emergency physicians. There are, however, a number of nerve blocks that are easy to learn, don’t require specialised equipment, and can be readily applied in EDs for minor procedures and longer acting forms of analgesia. Nerve blocks more applicable in the operating theatre or best done under ultrasound guidance are mentioned but not discussed in this article. This continuous professional development (CPD) article aims to provide guidance with respect to several key areas related to more commonly used types of regional anaesthesia in district level services. We discuss the importance of good clinical practice including thorough preparation of equipment and the patient to avoid common complications, clinical indications for regional blocks in the ED, local anaesthetic agents, different techniques for some common regional blocks, potential complications, and the need for a trained interprofessional team.

## Introduction

A regional block is a specific anaesthetic technique that is used to infiltrate a peripheral nerve with local anaesthetic (LA), thereby blocking nerve transmission to avoid or relieve pain.^1^ It is different from general anaesthesia (GA), which includes the triad of hypnosis and muscle relaxation and analgesia, as it does not affect the patient’s consciousness level to relieve pain.

Different types of regional anaesthesia (RA) may include local blocks, peripheral nerve blocks, nerve plexus blocks, intravenous RA and neuraxial (epidural and spinal) blocks. When effectively delivered, regional blocks can provide sufficient analgesia to carry out surgical procedures. Combining a local block and light sedation (such as ketamine) provides a good alternative to GA for minor procedures. They are also used to augment GA providing additional analgesia that can last hours to days.^2^ Nerve blocks are already commonly used in operating theatre environments and are now being used more frequently in emergency departments (ED) as an alternative to traditional methods of analgesia. This provides an accessible, safe and cost-effective alternative, particularly in low-resource settings such as sub-Saharan Africa, where safe and accessible surgical and anaesthetic care, is often limited.^3,4^ Adaptive leadership, training and adoption of regional techniques has lagged in developing countries despite it being a well-recognised option for over a decade with many potential benefits. In ED, musculoskeletal pain is a common and frequently neglected complaint. Regional blocks can safely be used for a variety of pathologies, sparing the use of opioids and shortening time to discharge in often overcrowded EDs.^5^ Additional advantages include avoidance of airway manipulation, reduced doses and side effects of systemic drugs, faster recovery time and significantly lower pain levels after surgery.^6^ Ultrasound-guided RA is not yet part of the formal training of emergency medicine registrars in South Africa, and certainly trained providers are very scarce in the district services. This continuous professional development (CPD) article aims to provide guidance on a few aspects of RA, addressing common concerns in primary care and district hospitals where anaesthesia is commonly provided by generalist medical officers rather than dedicated anaesthetic practitioners. This article will allow revision for generalist doctors on the different types and indications for plexus, peripheral and local nerve blocks (see Table 1a and 1b for the different types of RA). We will briefly describe important principles of practice, informed consent, preparation of the patient and equipment and a few common techniques used in primary care emergency settings. Frequently, equipment such as nerve stimulators and ultrasound are lacking in district hospitals, resulting in a reliance on anatomical landmarks. Advocacy for ultrasound-guided RA, which can improve access to safe and reliable anaesthesia in low-resource countries as it becomes more user-friendly, durable and affordable, should remain a target.^3^ We discuss the properties and maximum doses of LA drugs that are used, as well as recognising symptoms and signs of toxicity when using these drugs. We briefly discuss a failed block and other potential complications that may occur.

**TABLE 1a:** Scope of regional anaesthesia: Types and site of action of regional blocks.

Upper Limb Blocks	Site	Indications
Brachial Plexus Blocks (C5–T1): can be blocked at several sites	Interscalene block	Optimal for procedures on the shoulder, arm and forearm. Most intense C5–C7.
	Supraclavicular block	Optimal for humerus, elbow or hand surgery.
	Axillary plexus block	Optimal for elbow, forearm and hand procedures. Most intense C5–T1 (ulnar nerve).
Intravenous regional anaesthesia:	Bier Block	Used for short procedures on forearm, hand and even the leg. Provides good anaesthesia for skin but is often inadequate for bone. As regional techniques become more advanced, and ultrasound-guided blocks become more available, IV RA is no longer a preferred technique but can still be considered in low-resource settings.
Peripheral nerve blocks of the arm:	Median, radial and ulnar nerves blocks	Can be blocked at the wrist or the elbow. Used for minor hand and wrist procedures or to supplement brachial plexus blocks.
Local blocks:	Digital nerve block	Finger and toe blocks.

*Source*: South African Society of Anaesthesiologists. SASA guidelines for regional anaesthesia in South Africa 2016. Stellenbosch: South African Society of Anaesthesiologists; Morgan GE, Mikhail MS, Murray MJ. Clinical anaesthesiology. 4th ed. New York, NY: Lange; 2006

IV, intravenous; RA, regional anaesthesia.

**TABLE 1b:** Scope of regional anaesthesia: Types and site of action of regional blocks.

Lower limb blocks†	Site and/or indication
Psoas Compartment Block:	Lumbar plexus block within the psoas muscle. Optimal for hip, knee and femoral shaft surgery. Can be combined with a sciatic nerve block for foot, knee and ankle surgery.
Femoral Nerve Block and 3 in 1 Block:	Optimal for surgery to the anterior thigh, knee or femur. Does not reliably block the obturator nerve.
Sciatic Nerve Block:	Optimal for ankle and foot surgery, but when combined with a femoral nerve block, it can be used for knee and lower leg surgery.
Popliteal Block:	The sciatic nerve can be blocked at the popliteal fossa for ankle and foot surgery.
Ankle blocks:	Individual nerves can be blocked around the ankle – saphenous, sural and superficial peroneal nerves.
**Other blocks**	
Facial nerve blocks (supraorbital, infraorbital, mental), thoracic nerve blocks (intercostal, paravertebral), abdominal nerve blocks, pudendal and paracervical blocks and penile block.	-

*Source*: South African Society of Anaesthesiologists. SASA guidelines for regional anaesthesia in South Africa 2016. Stellenbosch: South African Society of Anaesthesiologists; Morgan GE, Mikhail MS, Murray MJ. Clinical anaesthesiology. 4th ed. New York, NY: Lange; 2006

RA, regional anaesthesia.

† , Consider neuraxial that may be simpler for lower limbs.

## Different types of regional anaesthesia used in theatre, emergency departments and for minor procedures (excluding neuraxial anaesthesia)

### Important principles for good practice

When doing any anaesthetic, it is important to always be well prepared. Good preparation, good clinical practice and adherence to national guidelines will decrease the incidence and severity of common and predictable anaesthetic complications. Difficulty can arise in low-resource settings where a reliable supply of electricity, pulse oximeters and oxygen is often lacking. However, as a basic standard of care in an ED setting, standard monitoring and functional resuscitation equipment and drugs are required. The skills and equipment to convert to a GA if required should always be in place.^7^ Not all generalists are comfortable with converting to GA. Adequately trained providers are, however, essential, an ongoing challenge where there is a high turnover of staff and a lack of training and support. Valid informed consent must always be obtained from the patient, advising them on the most appropriate technique that will depend on patient characteristics and experience of the clinician. Risks and benefits of the proposed technique and alternatives should be discussed. In the theatre setting, patients must be aware that a GA may still be required if RA is used as an adjunct or if an incomplete block occurs. The patient should be provided with enough information, covering risks and benefits, complications, expected duration of action of the block, how to care for the insensate limb (particularly minor blocks seen in the ED that are discharged), red flags, the socioeconomic setting and when to return to the clinic or district hospital.^7^ As a general rule, there are no clear-cut indications for one type of anaesthesia over another when either would be appropriate. Absolute contraindications should be excluded, such as known allergy to drugs or patient refusal. Bleeding diathesis is a relative contraindication, especially if the block is performed in an area where compression is possible. Active infection at the site of injection, pre-existing neurological deficit and poor patient cooperation are further relative contraindications. Anti-coagulation therapy is not a contraindication if stopped within the minimum recommended time before the block is performed.^7^ Thorough documentation should be done. Resuscitation equipment must be immediately available, and intravenous (IV) access established before the procedure. The practitioner must ensure the block is fully established, vital signs and physiological parameters are within normal limits and the patient is stable.^1^ Ensure an emergency trolley is immediately available for the management of complications and that the practitioner is skilled to rapidly recognise and treat these. Ensure appropriate measures are taken to minimise the risk of inadvertent nerve damage during the procedure. Always check and label drugs to be used to prevent error. In an ideal setting, a multidisciplinary team including nurses, doctors and clinical associates should work together to perform RA blocks. The team should be trained to prepare equipment and drugs, provide monitoring and have basic knowledge in evaluating the levels of pain and effects of RA.^1^ In the chaos of busy district casualties, there needs to be guidance as to which patients are good candidates for RA and which patients are not.

### Local anaesthetic agents and toxicity

Local anaesthetic drugs with or without adjuvants are used for RA. They are chosen according to their onset and duration of action, the degree of motor blockade and toxicity profiles. Lignocaine has a quick onset and short duration of action, and bupivacaine is longer acting.^1^ Local anaesthetic drugs produce transient loss of sensory, motor and autonomic function when injected or applied in proximity to neuronal tissue. Although LA is relatively free from side effects, accidental IV injection or excessive dose can lead to toxicity. The central nervous system tends to be affected first (perioral numbness and tinnitus followed by seizures and coma), followed by the cardiovascular system (severe bradycardia and dysrhythmias).^2^ Lipid emulsion (Intralipid) is used for bupivacaine toxicity, but is expensive, has a short half-life and is not readily available. Prolonged resuscitation may be required following bupivacaine toxicity. Cardiopulmonary resuscitation (CPR) should be continued for at least 60 min, as good neurological recovery can occur following LA systemic toxicity. The maximum dose of LA must not be exceeded.^1^

- Potency depends on lipid solubility and the ability to penetrate membranes and determines the lower limit of the dose required for nerve blockade while toxicity sets the upper limit.
- Onset of action is determined by pKa (LA are weak bases), concentration of the drug, lipid solubility and the connective tissue surrounding the nerve (infected tissue or abscesses create an acidic environment that delays the onset of LA).
- Duration of action is determined by lipid solubility, protein binding, dose and site of injection (plexus blocks last longer than subcutaneous and intrathecal blocks).^2,8^

### Dosing example

- Using 2% plain lignocaine in a 50 kg patient:
  ■ 50 kg × 4 mg/kg   = 200 mg maximum dose
             = 10 mL of 2% concentration
  ■ *ADD 10 mL 0.9% NaCl* = 20 mL of 1% concentration
  ■ *ADD another 20 mL of 0.9% NaCl* = 40 mL of 0.5% concentration.

A standard 2 mL dental cartridge (see Table 2)^7,8^ of lignocaine in South Africa contains 36 mg of lignocaine.

**TABLE 2:** Local anaesthetic dosing.

Drug	mg/mL	Max dose	Onset (duration)	Maximum volume (mL)			
				50 kg	60 kg	70 kg	80+ kg
Lignocaine 1%	10 mg/mL	4 mg/kg	± 5 min (30–90 min)	20	24	28	30 mL (300 mg)
Lignocaine 2%	20 mg/mL	-	-	10	12	14	15 mL (300 mg)
Lignocaine 1% *with Adrenaline*	10 mg/mL	7 mg/kg	± 5 min (60–180 min)	35	42	49	50 mL (500 mg)
Lignocaine 2% *with Adrenaline*	20 mg/mL	-	-	17.5	21	24.5	25 mL (500 mg)
Bupivacaine 0.5% ± *Adrenaline*	5 mg/mL	2 mg/kg	10–15 min (200+ min)	20	24	28	30 mL (150 mg)

*Source:* South African Society of Anaesthesiologists. SASA guidelines for regional anaesthesia in South Africa 2016. Stellenbosch: South African Society of Anaesthesiologists; Morgan GE, Mikhail MS, Murray MJ. Clinical anaesthesiology. 4th ed. New York, NY: Lange; 2006

Note: CMJAH ED Pain Management Protocol 2024.

### Equipment and preparation for the procedure

Equipment will depend on the technique used. The correct needle monitors as per the American Society of Anaesthesiologists’ standards for basic anaesthetic monitoring (pulse oximetry, electrocardiogram [ECG], blood pressure [BP]), IV access for rescue medications and possible sedation and supplemental oxygen should be available. An emergency trolley including emergency drugs (e.g., adrenaline, atropine and lignocaine), anaesthetic drugs (e.g., suxamethonium, propofol and ketamine), along with airway and intubation equipment, should be readily available to treat RA-related complications. An aseptic technique must be used for all blocks (sterile gloves, masks and surgical drapes).^1^

When performing a peripheral nerve block, the goal is to inject LA close to the nerve. Several methods have been used to identify the proximity of the needle to a nerve:

- Anatomical landmarks.
- Eliciting of paraesthesia caused by needle contact with the nerve.
- Electrical stimulation of a nerve without direct contact with the needle.
- Ultrasound-guided detection of the nerve.

In many public facilities at the district level in South Africa, nerve stimulators and ultrasound are not available, and there is a lack of training in the use of these techniques, and therefore, it will not be discussed in detail for the purpose of this article.

The role of ultrasound-guided RA is direct visualisation of the needle in relation to the nerve and other structures. Portable ultrasound machines are available, with high- and low-frequency probes, to identify both superficial and deep structures.^1^

Anatomical landmarks, combined with ultrasound guidance, can be used simultaneously to improve the success rate of the block, decrease the onset of action of the block, reduce the volume of LA required and reduce the risk of vascular puncture.

Each technique may be associated with specific complications. The main complications of RA are block failure, nerve injury and LA toxicity. Local anaesthetic toxicity and allergic reactions to LA drugs occur very rarely.^1^

When injecting LA, do not inject more than the maximum dose and be aware of the volume. Never inject under high pressure as the pressure effects could cause nerve damage. If there is pain or increased resistance, stop and reposition the needle. Always aspirate before injecting to avoid inadvertent injection into a vessel. If there is severe pain, stop and aspirate and the pain should stop immediately. You could be injecting directly into a nerve instead of around it. Allow 5 min – 10 min for maximal effect. Check on the sensory and motor effects depending on the block used. Warn the patient that they may feel numb for a few hours depending on the LA used.

Common blocks that can be used in the emergency departments and for minor procedures in district hospitals and clinics (see Table 3 for common limb blocks):

**TABLE 3:** Common limb blocks.

Scenario	Type of block	Possible indications	Anatomical landmarks and techniques
A 20-year-old man closed his finger in a door. The nail is avulsed, with a laceration to the nail bed. There is no fracture.	Digital nerve block^2,8^ Figure 1.	Procedures involving fingers or toes: Debridement Amputation laceration Nail bed injury Ingrown toenail Drainage abscess	Insert 25 G needle on the dorsolateral aspect of the affected digit just distal to metacarpophalangeal joint. Inject 2 mL – 3 mL 1% lignocaine as you withdraw. Repeat on the other side on the dorsomedial aspect. Do not use adrenaline for digital blocks.
	Wrist block^2,8,9^ Sites: Median Ulnar Radial Figure 2	Hand procedures: Carpal tunnel release Dupuytren’s contracture Trigger finger release Dislocations Drainage abscess Foreign body removal	Individual nerves can be targeted if surgery is limited to that cutaneous nerve distribution only. Wrist blocks give minimal motor blockade.
A 30–year-old woman presents with a laceration to the radial side of her palm after cutting an apple. There is no nerve or tendon injury.	Median nerve block^2,8,9^ Figure 3a and b	-	Found between tendons of flexor carpi radialis and flexor palmaris longus at proximal wrist crease. Ask the patient to flex the wrist and oppose the thumb and little finger. Insert 25 G needle at right angles to skin. A ‘pop’ is felt after the needle passes through the flexor retinaculum. If bone is contacted withdraw the needle by 2 mm – 3 mm. Inject 2 mL – 5 mL of LA at about 1 cm depth. On withdrawal infiltrate 2 mL – 3 mL subcutaneously to block the palmar cutaneous branch.
A 13-year-old boy presents with a dislocation of the proximal interphalangeal joint of his 5th finger on his left hand following a rugby game.	Ulnar nerve block^2,8,9^ Figure 3a and b	-	Blocked by inserting the needle medial to the ulnar artery and lateral to the flexor carpi ulnaris tendon at level of the wrist crease and directed towards the ulnar styloid. At a depth of 1 cm inject 2 mL – 4 mL LA. Dorsal branch of the ulnar nerve is blocked by injecting 5 mL subcutaneously around the ulnar aspect of the wrist.
A 60-year-old patient with burns on the radial aspect of the dorsum of the right hand requiring debridement.	Radial nerve block^2,8,9^ Figure 4	-	Only a sensory nerve at this stage lying subcutaneously on the dorsum of the wrist and dividing into branches. Easily blocked/field block requiring more extensive infiltration. Inject 5 mL subcutaneously about 1 cm proximal to the anatomical snuff box at the base of the thumb on the dorsal aspect. Aim the needle first medially and then laterally.
A 60-year-old woman presents with a fracture of femur, she is awaiting transfer to regional hospital and she is in a lot of pain.	Femoral nerve block^10,14^	Procedures on anterior thigh (skin graft) or knee or analgesia for hip/femur fractures in ED. Easy to perform as nerve superficial.	Stand on the side of the patient being blocked. Insert needle approximately 1 cm – 1.5 cm lateral to femoral artery, 1 cm – 2 cm distal to inguinal ligament, perpendicular to skin at midpoint inguinal ligament. Aim cephalad 3 cm – 4 cm, feel 2 pop’s. 20 mL bupivacaine usually sufficient. Aspirate after every 5 mL.
A 70-year-old woman presents following a fall on an outstretched hand. There is tenderness and swelling around her right wrist, with a ‘dinner fork’ deformity. X-ray confirms a Colles fracture.	Bier block (IV RA)^2,11,12^	Useful for cutaneous anaesthesia. Inadequate for bony surgery. Suitable only for short procedures of < 30 min. Used for: Manipulation Colles fracture Large lacerations Debridement of burns	IV LA is injected at the most distal venous portion of a lower or upper extremity. The anaesthetised limb has a tourniquet to avoid the spread of the anaesthetic agent to the systemic circulation. Only use lignocaine as accidental IV injection of bupivacaine has potential for significant systemic toxicity. Do not use adrenaline. Establish IV access in both arms, the 2nd line for safety. Dilute plain lignocaine to 0.5%. Double/single cuff tourniquet (depending on resources) is placed on the upper arm. Arm is raised above the patient’s head to exsanguinate it. The cuff is then inflated to 100 mmHg above the patient’s systolic BP. Check for the absence of radial pulse. If the double cuff system deflates the distal cuff, position the arm comfortably. The LA is injected IV into a distal vein on that hand, using 30 mL – 40 mL volume. 5 – 10 min for block to take effect. If after 15 min, the patient is experiencing tourniquet pain, the distal cuff can be inflated again over the area that is now anaesthetised and the proximal cuff deflated. The tourniquet may only be released completely 20 min after the anaesthetic was injected to avoid LA toxicity. Requires no specialised equipment. No residual analgesia therefore postoperative pain needs to be treated with systemic drugs.

*Source*: Please see the full reference list of the article for more information

IV, intravenous; ED, emergency departments; LA, local anaesthetic; RA, regional anaesthesia; BP, blood pressure.

**FIGURE 1:**
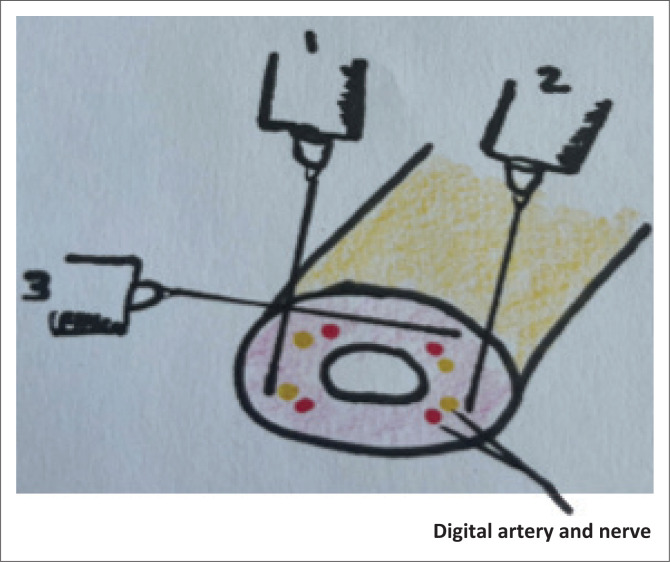
Digital nerve block.

**FIGURE 2:**
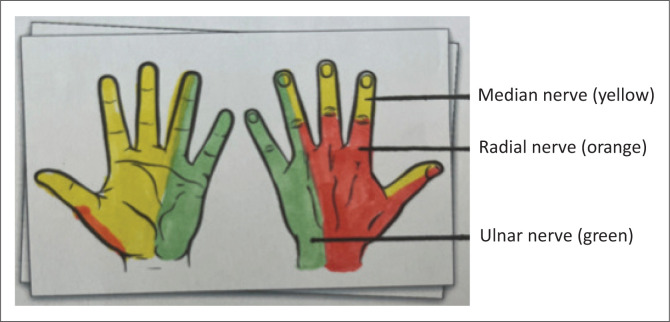
Nerve distribution in the hand.

**FIGURE 3:**
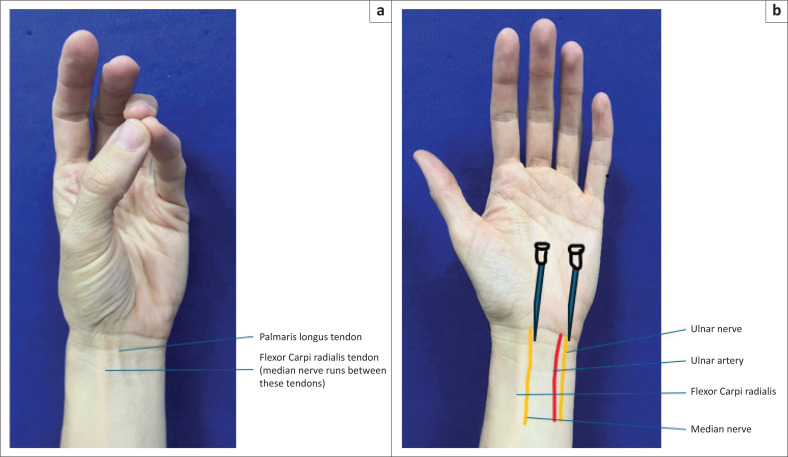
(a and b) Median and ulnar nerve blocks.

**FIGURE 4:**
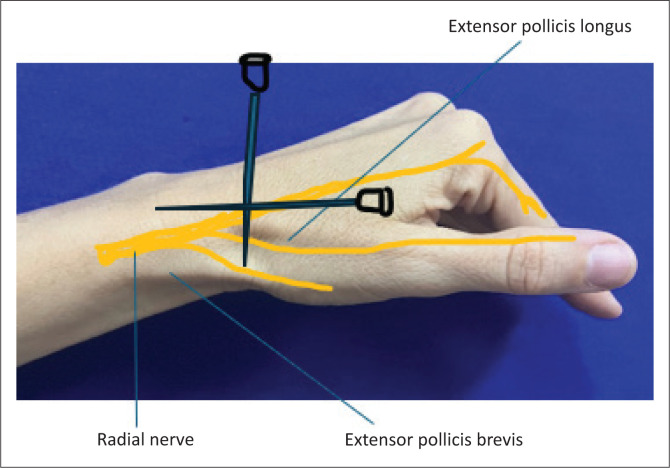
Radial nerve block.

### Dorsal penile nerve block

Used for circumcision or reduction of a paraphimosis.

A dorsal penile nerve block is typically achieved through a ring block at the base of the penis or a dorsal penile nerve block at the level of the pubic symphysis or a combination of both. It is an effective technique for RA using small volumes of LA.^13^

**FIGURE 5:**
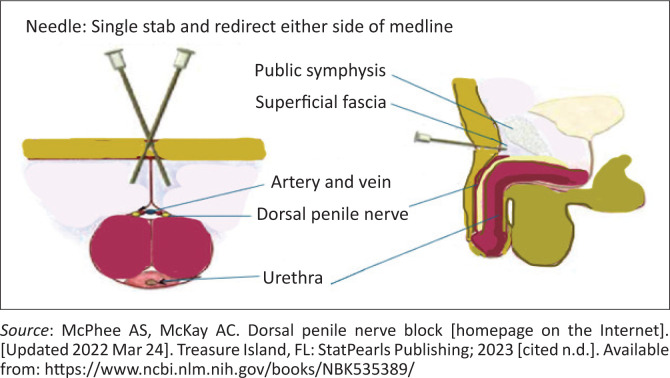
Dorsal penile nerve block.

For a dorsal penile nerve block, LA is injected into the bilateral spaces deep into the fascia on either side of the suspensory ligament. Insert a 25 G needle about 1 cm at the root of the penis on the dorsal aspect (at 01:30 and 11:30 positions) to penetrate Buck’s fascia and inject 5 mL on each side. Aspirate to prevent intravascular injection. Additional subcutaneous infiltration around the root of the penis (ring block) augments this block, as it covers cutaneous branches of the ilioinguinal and genitofemoral nerves. Always calculate the volume of anaesthetic required for circumcision in a child, which is much less.^13^ *Never ever use adrenaline for penile blocks* (Figure 5).^13^

## Conclusion

Safe RA is a useful clinical skill for district hospitals and clinics. It provides effective and often prolonged analgesia and improves patient satisfaction.^3^ Regional anaesthesia has wide application, with several nerve blocks that are easy to learn. Ultrasound-guided RA can broaden the scope, accessibility and safety of nerve blocks. Availability of context-sensitive guidelines and video demonstration should be made available in clinics and district hospitals.
